# Preparation of hollow magnetite microspheres and their applications as drugs carriers

**DOI:** 10.1186/1556-276X-7-210

**Published:** 2012-04-10

**Authors:** Francisco Márquez, Gloria M Herrera, Teresa Campo, María Cotto, José Ducongé, José M Sanz, Eduardo Elizalde, Óscar Perales, Carmen Morant

**Affiliations:** 1 School of Science and Technology, University of Turabo, Gurabo 00778, PR, USA; 2 Department of Chemistry, University of Puerto Rico, Mayagüez 00681, PR, USA; 3 Departamento de Física Aplicada C-XII, Universidad Autónoma de Madrid, Cantoblanco 28049, Madrid, Spain; 4 Department of General Engineering, University of Puerto Rico, Mayagüez 00681, PR, USA

**Keywords:** Fe3O4, Drug carrier, Rhodamine-B, Methotrexate

## Abstract

Hollow magnetite microspheres have been synthesized by a simple process through a template-free hydrothermal approach. Hollow microspheres were surface modified by coating with a silica nanolayer. Pristine and modified hollow microparticles were characterized by field-emission electron microscopy, transmission electron microscopy, X-ray diffraction, X-ray photoelectron spectroscopy, FT-IR and Raman spectroscopy, and VSM magnetometry. The potential application of the modified hollow magnetite microspheres as a drug carrier was evaluated by using Rhodamine B and methotrexate as model drugs. The loading and release kinetics of both molecules showed a clear pH and temperature dependent profile.

**Graphical abstract:**

Hollow magnetite microspheres have been synthesized. Load-release experiments with Rhodamine-B as a model drug and with Methotrexate (chemotherapy drug used in treating certain types of cancer) demonstrated the potential applications of these nanostructures in biomedical applications.

## Background

During the last few years, magnetic oxides particles [1-3] have attracted a great deal of attention due to their interesting applications in different fields such as catalysis [4,5], information storage [6], optoelectronics [7,8], and biomedical applications that include magnetic bioseparation, magnetic resonance imaging contrast enhancement, and targeted drug [9-18]. Among these magnetic materials, magnetic hollow structures with dimensions ranging from the nanometer to micrometer scale are of potential use for controlled release and drug delivery [19,20]. Up to date, syntheses including the use of templates are well known as very effective approaches to achieve hollow structures [21-28]. Nevertheless, these procedures involve tedious synthesis steps and normally have economic drawbacks. Over the past recent years, different template-free synthesis methods have been tested with very interesting results. Thus, Zhu et al. have synthesized monodisperse magnetite hollow microspheres with diameters ranging from 200 to 300 nm and shell thickness of ca. 50 nm by using a free-template solvothermal procedure [29]. In a recent paper, we synthesized monodisperse hollow magnetite microspheres by a one-step process through a template-free hydrothermal approach employing FeCl_3 _and ferrocene as precursor and propylene glycol-isopropanol as solvent [20]. These materials are characterized by having a large surface area, a very low density, and also a strong magnetic response that make them interesting candidates to be used as drug carriers. The internal hollow spaces may be used as hosts for the encapsulation of guest molecules or specific drugs. However, the use of these nanostructured materials has some limitations arising from the tendency to aggregation because of their high specific area and strong interparticle interactions [30]. To overcome this drawback, different strategies for the chemical stabilization of the naked hollow magnetite have been tested, including the incorporation of polymer structures on the magnetite surface or the amine functionalization of the hollow magnetite surface [31,32].

**Figure 1:**
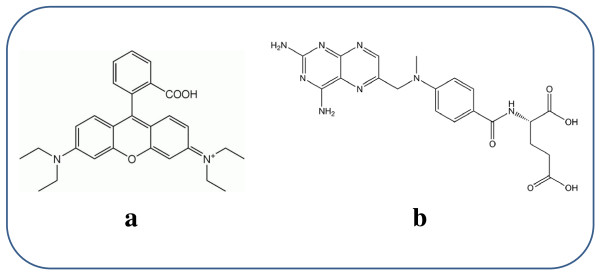
**Structure of Rh-B (a) and MTX (b)**.

In the present work, we have synthesized monodisperse hollow magnetite microspheres by using a one-step solvothermal procedure. To increase the solubility in polar solvents in avoiding aggregation, these microspheres were surface modified by growing SiO_2 _nanolayers via sol-gel process. Primary (hollow magnetite microspheres) and modified microspheres (SiO_2_@hollow magnetite microspheres) have been used to encapsulate and to study under different conditions the release of two test compounds (Rhodamine B (Rh-B) and methotrexate (MTX)), a chemotherapy drug used in treating certain types of cancer), see Figure 1. The release of both compounds has been shown to depend on the temperature and pH value of the environment.

## Methods

All reagents used in the present investigation were of analytical grade. FeCl_3 _anhydrous (99.98%), propylene glycol and diethyl amine were provided by Fisher Scientific (Pittsburgh, PA, USA) and used as received. Rhodamine B and methotrexate were provided by Merck Chemicals (Beeston, Nottingham, UK) and used without further purification. Pure ethyl alcohol and tetraethyl orthosilicate (TEOS) were provided by Aldrich Chemical Co. MilliQ (St. Louis, MO, USA)water (18.2 MΩ.cm at 25°C) was used for all experiments.

### Synthesis of hollow magnetic Fe_3_O_4 _microspheres

For a typical synthesis, 2 mmol of FeCl_3 _were dissolved in 10 mL of propylene glycol; the solution was magnetically stirred at room temperature for 10 min followed by soft ultrasonic treatment for 5 min. Next, 1 mmol of ferrocene was added, and the solution was stirred at room temperature for 2 h. Finally, 3 mL of diethyl amine were added to the mixture. The solution was placed into the Teflon-lined stainless steel autoclave of 30 mL capacity and maintained at 180^°^C for 24 h. Lower synthesis temperatures (100 to 150^°^C) were tested although the synthesized hollow magnetite microspheres were not stable, and the degradation was produced in a few days, generating compact magnetite nanoparticles of no more than 10 nm diameter. The use of FeCl_3_/ferrocene as precursor is required to induce a faster self-assembling of nanoparticles into hollow microspheres.

After cooling to room temperature, the black sediment is collected and washed in water by five centrifugation (6,000 rpm, 10 min)-redispersion cycles. Next, the black sediment was resuspended in ethanol and washed by two centrifugation (6,000 rpm, 5 min)-redispersion cycles. Finally, the microspheres were suspended in ethanol and dried overnight at 60°C. Hollow Fe_3_O_4 _microspheres were maintained in sealed containers before characterization.

### Preparation of magnetic SiO_2_@Fe_3_O_4 _microspheres

**Figure 2:**
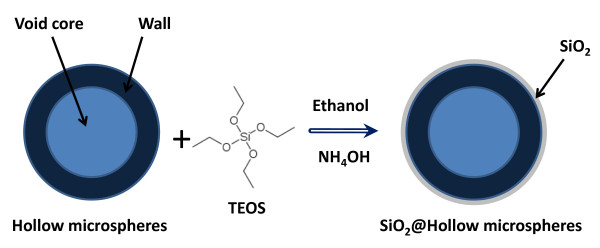
**Schematic representation of the synthesis of SiO_2_@hollow magnetite microspheres**.

The schematic procedure used to synthesize SiO_2_@Fe_3_O_4 _hybrid hollow microspheres is illustrated in Figure 2, and the detailed synthesis is described as follows.

The silica shell has been prepared by using a modified Stobe-Fink-Bohn method [33,34], which consists of two steps: (i) the hydrolysis of TEOS (Si(C_2_H_5_O)_4_) in ethanol, in presence of ammonium hydroxide as catalyst, and (ii) the polymerization phase, where the siloxane (Si-O-Si) bonds are formed and anchored on the hollow magnetite surface. In a typical synthesis, 50 mg of hollow Fe_3_O_4 _microspheres were added to 20 mL of ethanol. The mixture was homogenized in an ultrasound bath for 10 min. Next, 0.5 mL of deionized water and 0.5 mL of ammonium hydroxide (36%) were added into the flask under vigorous mechanical stirring to prevent particles from settling. Temperature was termostatized at 20^°^C for at least 20 min, and after this period, 0.5 mL of TEOS was added dropwise to the reaction mixture over the course of 5 min under constant stirring in fumehood. After addition, reaction mixture was vigorously stirred for 30 min. Next, the solvent of the reaction mixture is evaporated at 60^°^C overnight. The residue is washed twice in distilled water and finally in ethanol and allowed to dry in vacuum at room temperature.

### Encapsulation efficacy within hollow microparticles

To evaluate the potential application of these hollow spheres as drug carriers, three different infiltrations of an organic compound were tested. Rh-B was chosen as test molecule for different reasons. First of all, the size of this molecule is not too small (as occurs with the most of drugs used for therapeutic treatments). On the other hand, this compound is soluble in polar solvents and shows a very high fluorescence quantum yield that can be useful to evidence the presence of very low amounts of this compound, even at trace levels. In this way, Rh-B at three different concentrations (0.05, 0.1, and 0.2 mg/mL) in ethanol were prepared. The infiltration consisted in adding the appropriate amount of Rh-B solution into an erlenmeyer flask containing 5 mg of hollow particles. This mix was mechanically stirred, and the result for each Rh-B concentration was evaluated at different time period, namely 1, 2, 4, 6, 8, 9, and 12 h. These infiltrations were developed at 20^°^C and 40^°^C. After infiltration, the particles were centrifuged (1,000 rpm, 5 min) and washed three times with MilliQ water. The particles loads with Rh-B were dried overnight at 60^°^C. To determine the amount of Rh-B storage in the hollow microspheres, thermogravimetry (TG) was employed to directly measure the weight loss of as-prepared product.

The release kinetics was studied in aqueous solution by controlling the pH and temperature of the solvent by the dialysis bag method. The dialysis bag was soaked in water for 3 h before use. The dialysis bag retained the magnetite microspheres allowing free Rh-B to diffuse into the solution of study. To monitor the Rh-B release by pH and temperature effect, solutions at different times were analyzed by fluorescence spectroscopy. The pH of solution was adjusted using acetate 0.01 M (pH = 3.7) and phosphate 0.01 M (pH = 7.4) buffers. All load and release tests developed on Rh-B were also tested on MTX, a drug used in some cancer treatments.

### Characterization

X-ray powder diffraction patterns (XRD) were collected using an X'Pert PRO X-ray diffractometer (PANalytical, The Netherlands) in Bragg-Brentano goniometer configuration. The X-ray radiation source was a ceramic X-ray diffraction Cu anode tube type Empyrean of 2.2 kW.

Raman spectra were collected using a micro-Raman Renishaw RM2000 single grating spectrograph, equipped with 532 and 785 nm excitation sources. Raman spectra were acquired in the spectral range of 3,200-100 cm^-1^. The acquisition time for each measurement was 20 s and a defocused laser power level in the range of 10 to 60 mW was used to prevent the possible thermal effects on the samples. Infrared spectra were obtained in the 4,000-400 cm^-1 ^region by using a Bruker Optics IFS 66 series FT-IR spectrometer (Bruker Optik Gmbh, Ettlingen, Germany). The variation in magnetization and coercivity of the hollow magnetite samples was determined by using a Lake Shore-7400 vibrating sample magnetometer (VSM) (Lake Shore Cryotronics Inc, Westerville, OH, USA) at room-temperature.

Field emission scanning electron microscopy (FE-SEM) images were obtained using a JEOL JM-6400 microscope. High Resolution Transmission electron microscopy (HRTEM) images were recorded on a JEOL 3000 with an acceleration voltage of 300 kV.

X-ray photoelectron spectroscopy (XPS) measurements were performed on an ESCALAB 220i-XL spectrometer (VG-Scientific, East Grinstead, UK, by using the non-monochromated Mg Ka (1,253.6 eV) radiation of a twin-anode, operating at 20 mA and 12 kV in the constant analyzer energy mode, with a PE of 50 eV. In order to remove charging shifts and deal with Fermi edge coupling problems, binding energies were corrected using the peak of the C-(C, H) component coming from contamination carbon (set to 284.6 eV). The samples were pressed on to a molybdenum support in an argon-filled glove box and then were put into the preparation chamber to pump for approximately 24 h at 60^°^C under a pressure of about 10^-7 ^Pa to minimize surface contamination. Small amounts of activated carbon fine powder were added to the samples to improve their conductivity. The vacuum during spectra acquisition was better than 5 × 10^-9 ^mbar.

TG analysis data were obtained with a TGA Q-500 instrument (TA Instruments)under inert atmosphere of nitrogen at a heating rate of 20^°^C min^-1^, from 100 to 600^°^C. The specific surface area, the pore volume, and the pore size distribution of the hollow magnetite microspheres, were measured using a Micromeritics ASAP 2020. The micropore volume, WMP [cm^2^/g], was measured using the Barrett-Joyner-Halenda (BJH) approach [35].

## Results and discussion

### Characterization of magnetite microspheres

**Figure 3:**
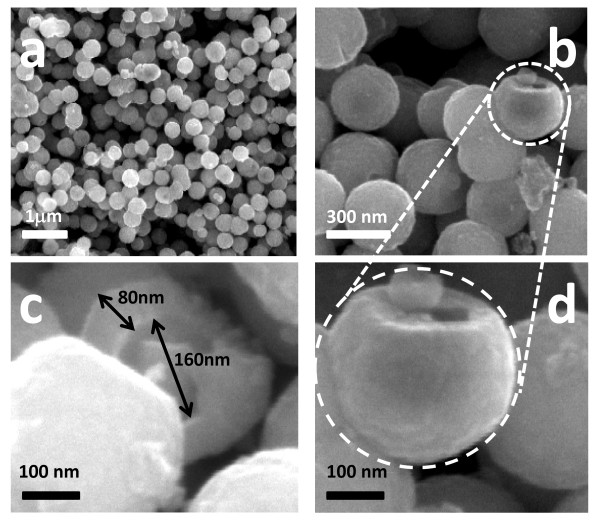
**FE-SEM images of the as-synthesized hollow magnetite microspheres obtained at different magnification**.

Figure 3a,d gives the representative FE-SEM micrographs of as-synthesized magnetite microspheres. As can be seen there, these microparticles are very regular in size with diameters of ca. 300 nm and high sphericity. Figure 3c,d shows some broken spheres confirming that these particles have a hollow interior. In all cases, magnetite microspheres show a mean inner hole of around 160 nm and wall thicknesses of ca. 80 nm. As stated in a previous work, these dimensions are directly related to the synthesis temperature and time of growth [20]. In our research, it was found that the hydrothermal synthesis at 180^°^C and 24 h provided the best results. Additionally, magnetite microspheres obtained under these reaction conditions showed high stability even in water solution.

**Figure 4:**
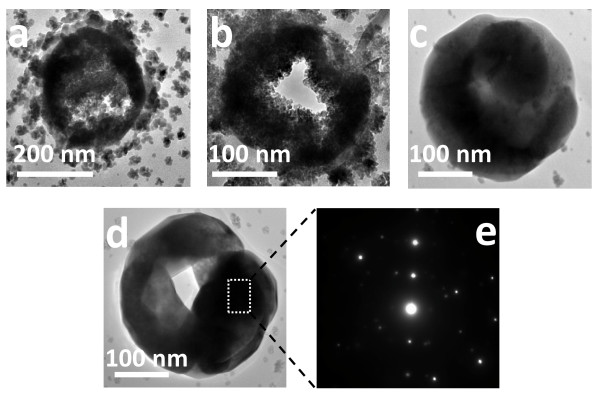
**TEM images of the hollow magnetite microspheres obtained at different reaction times**. TEM images of the hollow magnetite microspheres obtained at different reaction times: 8 h (a), 16 h (b) and 24 h (c, d). Figure 4e corresponds to the SAED pattern of the boxed region shown in image d.

These structures have been further investigated by TEM. Figure 4 shows the morphological characterization of magnetite microspheres obtained after different growth steps. As can be seen, the size of the spheres increases from diameters around 200 nm in initial growth steps (Figure 4a) to ca. 300 nm (after 24 h). Figure 4a,b shows that these spheres are built from smaller building blocks of no more than 5-8 nm assembled together to form the shell wall. The final structures, synthesized after a reaction time of 24 h, show a homogeneous surface with constant dimensions. The contrast between the black margin and the clear center of the microparticles confirms the existence of hollow spheres, according to the FE-SEM observations. Additionally, the corresponding selected area electron diffraction (SAED) (Figure 4e) taken from an individual hollow microsphere, as shown in Figure 4d (marked by a rectangle), reveals the crystalline nature of the material.

**Figure 5:**
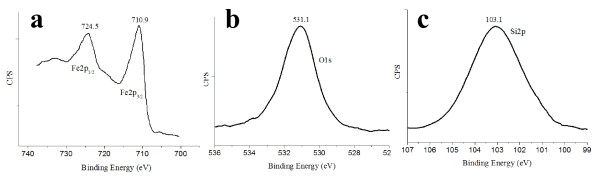
**XPS analysis of the magnetite microspheres in the Fe2p (a), O1s (b), and Si2p (c) regions**.

Different mechanisms have been proposed for the synthesis of hollow magnetite microspheres [3,36-39]. During the hydrothermal synthesis, the propylene glycol can act as both high-boiling point solvent and reducing agent, producing the partial reduction of Fe^3+ ^to Fe^2+^. The diethyl amine provides the pH required for the hydroxylation of iron species as Fe(OH)_2 _and Fe(OH)_3 _and after that, a dehydration process of both species is probably the mechanism that finally generates the formation of small particles of Fe_3_O_4_. This process is produced forming small aggregates of Fe_3_O_4 _nanoparticles that experience effects driven by a minimization of the surface energy arising finally to the formation of microspheres with internal voids. Nevertheless, additional research is still required to understand the precise mechanism of transformation of small aggregates of Fe_3_O_4 _particles into hollow structures. Figure 5a,b shows the Fe2p and O1s regions, corresponding to the as-synthesized magnetite microspheres. As can be seen in Figure 5a, Fe2p has binding energy (BE) values at 710.9 ev (Fe2p_3/2_) and 724.5 eV (Fe2p_1/2_). The BE values along with the absence of charge transfer satellite near to the Fe2p_3/2 _peak are consistent with the presence of mixed oxidation states of iron such as Fe_3_O_4 _[40,41]. Additionally, the XPS profile corresponding to the O1s region (Figure 5b) is characterized by only one peak at ca. 531.1 eV that can be attributed to O-Fe in magnetite phase. These assignations agree with the literature and evidence that the only phase present in the samples is Fe_3_O_4_.

**Figure 6:**
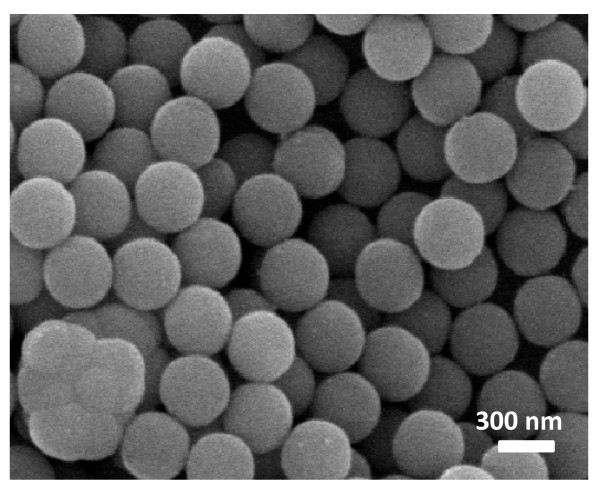
**FE-SEM image of the hollow magnetite microspheres after coated by a silica nanolayer**.

The Fe/O atomic ratio (as determined by XPS) was 0.71 being in agreement with the expected value for magnetite (0.75). To increase the solubility in polar solvents in avoiding aggregation, magnetite microspheres have been surface modified by growing SiO_2 _nanolayers via sol-gel process. As shown in Figure 5c, only one peak (103.1 eV) could be observed in the Si2p region that was assigned to SiO_2_. As can be derived by comparing the FE-SEM images of Figure 3, corresponding to hollow magnetite microspheres, with that obtained after depositing a silica layer on the surface (Figure 6), the surface texture is apparently more homogeneous showing diameters slightly increased (ca. 20 nm).

**Figure 7:**
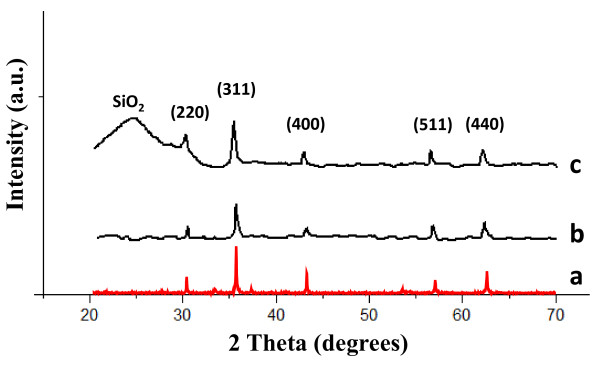
**The XRD patterns of a magnetite standard sample**. XRD patterns of a magnetite standard sample (a), pristine hollow magnetite microspheres (b) and silica coated magnetite microspheres (c).

Figure 7 shows the XRD patterns of as-synthesized hollow magnetite microspheres (Figure 7b) and SiO_2_@hollow magnetite microspheres (Figure 7c), along with XRD standard pattern for magnetite (Figure 7a), for comparison purposes. The (220), (311), (400), (511), and (440) reflections have been indexed to the inverse cubic spinel structure of Fe_3_O_4 _(JCPDS 85-1436). The broad diffraction band observed in the range of 20 < 2t < 32 (Figure 7b) corresponds to the amorphous or nanocrystalline silica of the microspheres surface layer.

**Figure 8:**
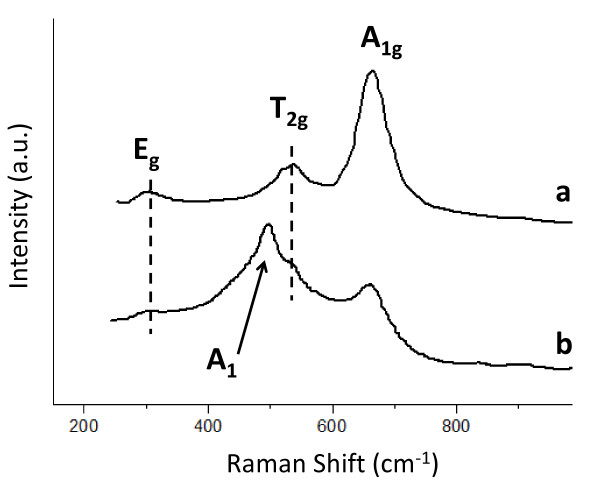
**The Raman spectra of the as-synthesized hollow magnetite microspheres**. Raman spectra of the as-synthesized hollow magnetite microspheres (a) and after growing a silica nanolayer on the surface (b).

The structural phase of hollow and SiO_2_@hollow magnetites has also been studied by Raman spectroscopy. Figure 8 shows the Raman spectra of both types of materials. As can be seen there, hollow magnetite microspheres (Figure 8a) show three Raman peaks at ca. 669 cm^-1^, 540 cm^-1^, and 300 cm^-1 ^that have been assigned to the active modes A_1g_, T_2g_, and E_g _[42-44]. Once the hollow magnetites have been coated with a silica layer, a new Raman peak at ca. 496 cm^-1 ^appears. This vibration has been assigned to the A_1 _active mode, characteristic of Si-O bonds.

**Figure 9:**
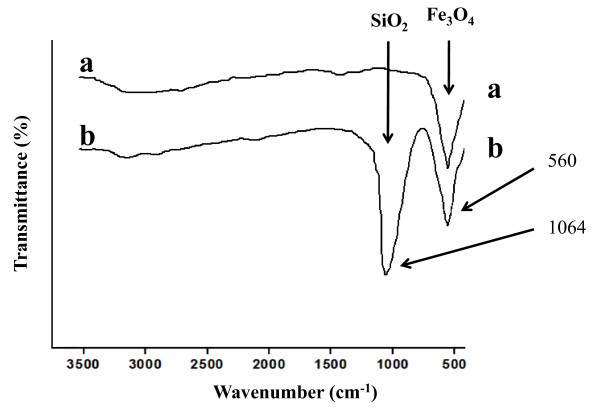
**FT-IR spectra of the as-synthesized hollow magnetite microspheres (a) and silica coated magnetite microspheres (b)**.

The FT-IR spectra of as-synthesized hollow magnetite microspheres (Figure 9a) and SiO_2_@hollow magnetite microspheres (Figure 9b) are characterized by a strong absorption band at ca. 560 cm^-1 ^attributed to the typical band of Fe_3_O_4_, corresponding to the stretching vibration modes of Fe-O [42,45]. After coating by a silica layer, a new band appeared at about 1,064 cm^-1 ^(Figure 9b) being assigned to stretching of Si-O-Si bands at the surface of the SiO2@hollow magnetite microspheres.

**Figure 10:**
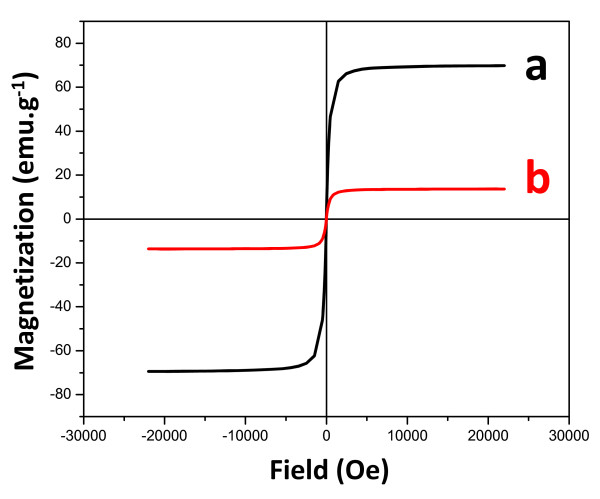
**The magnetization curves obtained at 25^°^C of hollow magnetite microspheres**. Magnetization curves obtained at 25^°^C of hollow magnetite microspheres (a) and silica coated magnetite microspheres (b).

The magnetic properties of the synthesized materials were also investigated by VSM magnetometry under room-temperature conditions. The corresponding hysteresis loops in the -30 + 30 kOe range are shown in Figure 10. The saturation magnetization (M_s_) values were 69.6 emu g^-1 ^(hollow magnetite) and 13.7 emu g^-1 ^(SiO_2_@hollow magnetite). The observed saturation of the magnetization profiles and the lack of coercivity and remanence were expected for ferrimagnetic magnetite particles [46]. The difference in Ms observed when hollow spheres are coated by silica can be attributed to the non-magnetic contribution of diamagnetic silica onto the magnetite surface. However, specific interactions between the silica surface layer and the magnetite nanoparticles forming the hollow structure could not be ruled out at present. More detailed investigations concerning this deleterious effect of silica on the magnetization of the hollow structures are currently in progress. The absence of coercivity in the hollow magnetite structures could also suggest a superparamagnetic behavior that could be ascribed to the fact that magnetite microspheres are built by many small nanoparticles, as stated by TEM, which show oriented aggregation into a hollow structure [47]. Mossbauer analyses could provide additional insights about the probable coexistence of superparamagnetic particles in as-synthesized hollow structures.

Hollow magnetite microspheres can easily be dispersed in water even though the sedimentation is produced in a short time (typically no more than 15 min). Silica-coated hollow magnetite microspheres previously dispersed in water form a permanent suspension that can be separated from the solvent by applying an external magnetic field. The magnetic particles can be brought back into the original solution by removing the external magnetic field and gently shaking the solution.

The Brunauer-Emmett-Teller surface area and pore parameters of the synthesized samples were determined by N_2 _adsorption-desorption isotherm measurement at 77 K. Pristine hollow microspheres have a surface area of ca. 50 m^2 ^g^-1^, while silica-coated hollow microspheres show a slightly lower 46 m^2 ^g^-1^. The pore size distributions were determined using the Barrett-Joyner-Halenda calculations [35] on the desorption portion of the isotherm at 77 K revealing a narrow distribution centered at 3.2 and 2.4 nm for pristine and coated microspheres, respectively. This result agrees with the fact that microspheres are composed by smaller nanoparticles organized to form hollow spheres allowing the existence of pores with nanometric dimensions.

### Loading and release of test molecules

**Figure 11:**
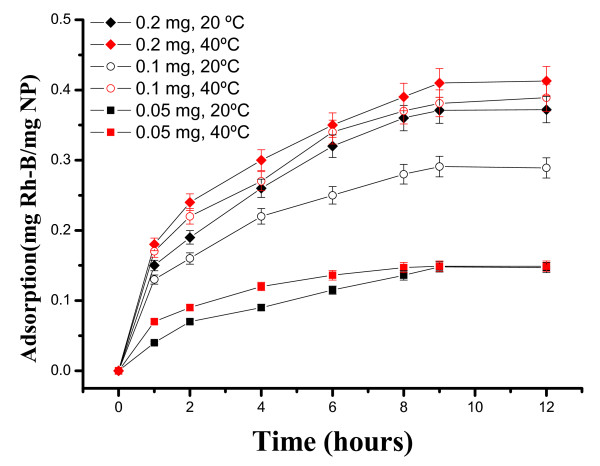
**Loading kinetics of Rh-B in SiO_2_@hollow magnetite microspheres at different Rh-B concentrations and temperatures**.

To investigate hollow magnetite microspheres as a candidate of drug carriers for delivery, we selected Rh-B and MTX as models. MTX is a well known chemotherapy drug used in treating certain types of cancer. Figure 11 shows the loading of Rh-B in SiO_2_@hollow magnetite microspheres at different Rh-B concentrations (0.05, 0.1, and 0.2 mg/mL) and temperatures (20^°^C and 40^°^C). As expected, the microspheres show distinctly different loading capacity toward Rh-B at different temperatures. As can be seen, the highest loading capacity is observed at higher temperature (40^°^C) and clearly depends on the initial Rh-B concentration. As determined by TG, the loading of Rh-B in SiO_2_@hollow magnetite microspheres is ca. 0.413 mgRh-B/mgMP at 40^°^C and after contact times of 9 h.

**Figure 12:**
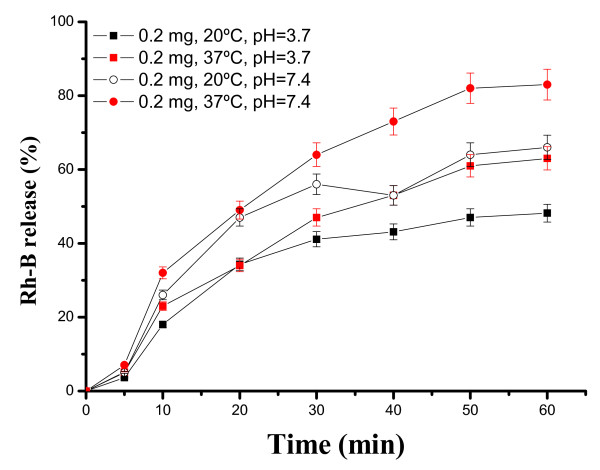
**Release kinetics of Rh-B incorporated within SiO_2_@hollow magnetite microspheres at different pH and temperatures**.

The release behavior of Rh-B from SiO_2_@hollow magnetite microspheres was examined in buffered solutions at pH = 3.7 and 7.4, and the results are shown in Figure 12. As can be seen there, the higher release values are observed near to physiological conditions in the human body (pH = 7.4 and 37^°^C). Releasing of Rh-B from the host is produced in a short period of time, typically during the first hour and clearly depends on pH and temperature.

**Figure 13:**
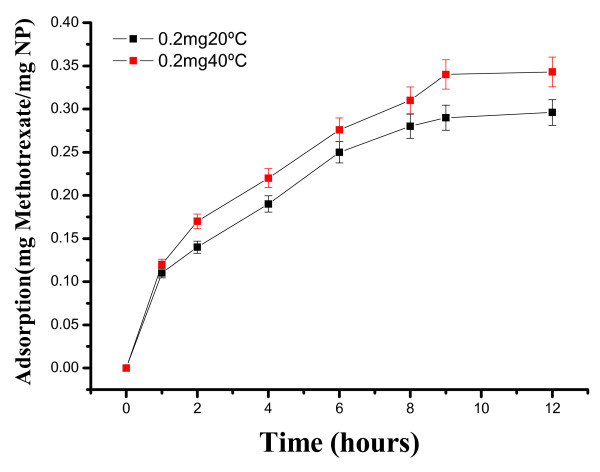
**Loading kinetics of MTX in SiO_2_@hollow magnetite microspheres at 20**^°^**C and 40**^°^**C**.

**Figure 14:**
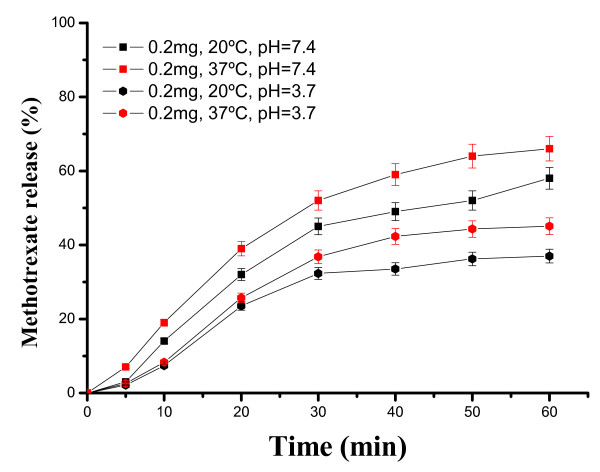
**Release kinetics of MTX incorporated within SiO_2_@hollow magnetite microspheres at different pH and temperatures**.

In a similar way, we examined the loading and release kinetics of MTX in SiO2@hollow magnetite microspheres. As shown in Figure 13, the loading of MTX depends on the temperature, and the maximum value is observed after 9 h of contact time (0.343 mgMTX/mgMP at 40^°^C). The decrease of MTX loading in SiO_2_@hollow magnetite microspheres, as compared with loadings of Rh-B, can be ascribed to the reduced solubility of MTX in water. Nevertheless, the loadings of MTX were surprisingly higher than those expected, exceeding our expectations. The release kinetic of MTX was studied after incubating in phosphate (pH = 7.4) and acetate (pH = 3.7) buffer solutions at two different temperatures (20^°^C and 37^°^C). As shown in Figure 14, MTX was released more rapidly (typically during the first 60 min) at pH = 7.4 and 37^°^C). These results show that within the first hour almost 70% of MTX was released from the microparticles, and the remaining drug was slowly released in a sustained fashion over a period of 5 h (not shown). Such long stability and delay of MTX could be due to favorable ionic interactions between amino and carboxylic groups of MTX and the surface of the magnetite nanoparticles forming the hollow microparticles. Additional work is currently in progress to provide answers to these outstanding issues.

## Conclusions

In the present work, we have synthesized hollow magnetite microspheres by a simple one-step hydrothermal procedure. With the aim to increase the solubility in polar solvents, these microspheres were subsequently surface modified by growing a silica nanolayer via sol-gel process. The potential application of the modified hollow magnetite microspheres as a drug carrier was evaluated by using Rh-B and MTX as model drugs. The loading and release kinetics of both molecules experienced a pH and temperature dependent profile. It is expected that this dependency could be modified and selected for specific functions, opening up promising applications in biomedical fields.

## Competing interests

The authors declare that they have no competing interests.

## Authors' contributions

FM, GMH, MC, JD, synthesized different samples. FM, CM, TC, JMS, OP, and EE characterized the synthesized samples by Raman, XPS, FE-SEM, VSM, and TEM. All authors read and approved the final manuscript.
